# Influence of Lip Appearances and Tooth Shade on Smile Attractiveness Perception

**DOI:** 10.1155/2022/5952643

**Published:** 2022-12-16

**Authors:** Nantawan Krajangta, Panupat Phumpatrakom, Chayaporn Supachartwong, Pornpisut Kamolwarin, Seelassaya Leelaponglit

**Affiliations:** ^1^Division of Restorative Dentistry, Faculty of Dentistry, Thammasat University, Pathumthani, Thailand; ^2^Thammasat University Research Unit in Restorative and Esthetic Dentistry, Thammasat University, Pathumthani, Thailand; ^3^Division of Endodontic Dentistry, Faculty of Dentistry, Thammasat University, Pathumthani, Thailand; ^4^Faculty of Dentistry, Thammasat University, Pathumthani, Thailand

## Abstract

This study determined the effect of lip thickness, lipstick color, and tooth shade on the smile attractiveness perceptions of dentists, laypersons, dental students, and other faculty students. A set of 27 smile photographs was prepared with different lip thicknesses (Tk, thick; *M*, medium; and Tn, thin), lipstick shade (*R*, red; *P*, pink; and *O*, orange), and tooth shades (0*M*1, 0*M*3, and *A*1). A total of 212 Thai participants in four rater groups (dentists, laypersons, dental students, and other faculty students) rated smile attractiveness using a visual analog scale (VAS). Statistical analyses were performed using the Kruskal-Wallis test and pairwise analysis at a 0.05% level of significance. Tk or *M* lip thickness was associated with more smile attractiveness than Th lip thickness. The *R* lipstick is more attractive than the *P* and *O* lipsticks. The 0*M*1 tooth shade appeared to be the most attractive for laypersons and other faculty students, whereas tooth shades (0*M*1, 0*M*3, or *A*1) did not influence the smile attractiveness perception of dentists and dental students. The smile attractiveness perception was influenced by the lip appearance and tooth shade for each rater group, which are essential for an attractive smile design.

## 1. Introduction

The perception of smile attractiveness is the level at which the features of a smile are perceived as esthetic, pleasing, and beautiful. Smile attractiveness is a key factor in increasing self-esteem and personal attractiveness, creating a good first impression on people, and catching their eye like a spotlight on the red carpet [1, 2]. The smile display zone is composed of several components, including teeth, gums, and lips, which influence the perception of smile attractiveness [3–5]. In general, healthy teeth and gums and good shape and alignment of teeth create an attractive smile [4, 6, 7]. Many people visit dentists to improve their smile for a more attractive appearance. Smile design with strategic consideration of smile components is an important step in planning to achieve an attractive smile [4, 6]. Lipstick is a popular makeup tool that helps women enhance their smile's appearance [8–10]. Hence, lip characteristics, including lip thickness and lipstick colors, should be considered during tooth shade selection to achieve an attractive smile. People choose a brighter tooth shade for a more attractive smile [3, 11, 12]. Brighter tooth shades in dental restorations have several levels of shade [3]. This makes it difficult for dentists and patients to choose the appropriate tooth shade of restorative materials to create an attractive smile.

The influence of lip thickness, application of a lipstick, and tooth shade on smile attractiveness perception has not been assessed. In addition, there is no study on the effects of different personal backgrounds and experiences on valuing the appeal of a smile [1, 5, 13]. Thus, this study aimed to assess smile attractiveness perception according to differences in lip thickness, lipstick color, and tooth shade among different rater groups, including dentists, laypersons, and dental (3^rd^–6^th^ years) and other faculty (1^st^–4^th^ years of nondental) undergraduate students. The present study collected data via a photographic questionnaire using a 10-cm visual analog scale (VAS) to analyze the attractiveness of a smile, as used in previous studies [14, 15]. The null hypothesis of this study was that there is no significant difference in smile attractiveness perception according to lip thickness, lipstick color, and tooth shade, as evaluated by all participants and each rater group (dentists, laypersons, and dental and other faculty students).

## 2. Materials and Methods

### 2.1. Participants

This study used a cross-sectional photographic questionnaire and was approved by the Human Research Ethics Committee of Thammasat University (science; HREC-TUSc, project no. 040/2565) prior to commencement. A minimum sample size of 212 participants was estimated using the *G*-power program, with an effect size of 0.25, an alpha error of 0.05, and a power size of 0.80. We collected data from 212 Thai individuals aging from 18 to 60 years, who used the Thai language. Both male and female participants with the purpose of sampling four rater subgroups (*n* = 53) including dentists, laypersons, and dental (3^rd^–6^th^ years) and other faculty (nondental) undergraduate students were included. All participants were recruited from Thammasat University, Pathumthani, Thailand. Participants who reported of not being color blind (determined using the Ishihara test) and denied having eye or vision problems were included in this study. Participants with a visual impairment or color blindness were excluded from the study.

### 2.2. Questionnaire Preparation

A Thai digital questionnaire was prepared, which was divided into three parts: an introduction with brief information regarding the study; Part 1, which included demographic information (gender and rater group); and Part 2, which included the photographic questionnaire for rating smile attractiveness.

**Figure 1:**
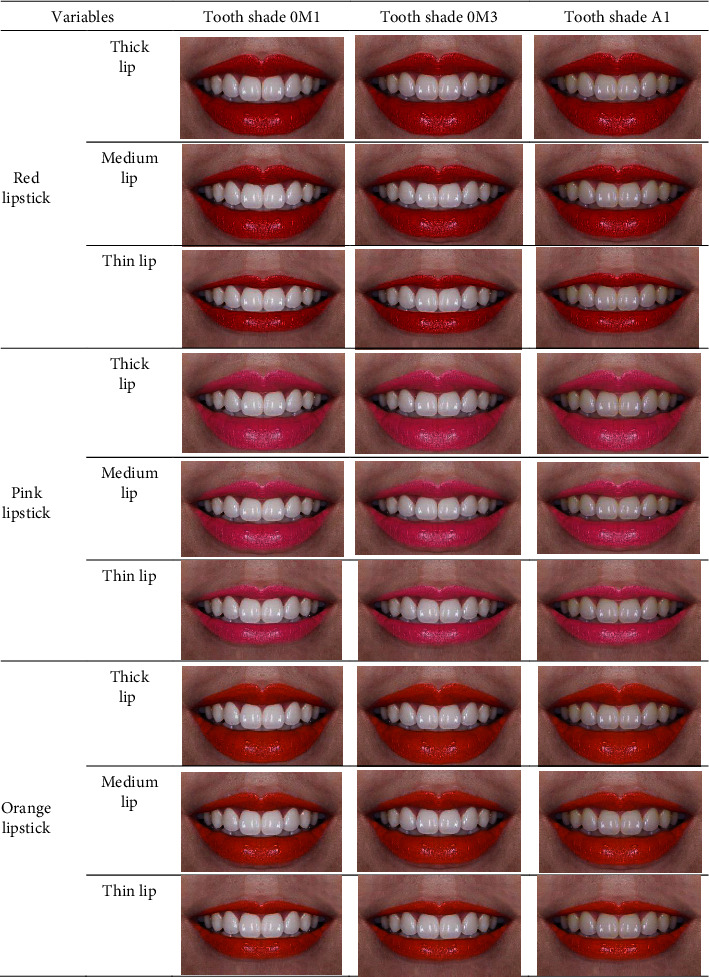
The photographic questionnaire with 27 photographs of various lip thicknesses, lipstick shades, and tooth shades.

A photograph of a woman's smile was taken using a digital camera (D7100 with lens macro 105 and flash r1c1; Nikon Corporation). The smile photograph was digitally altered using Adobe Photoshop (Adobe Systems Inc., San Jose, CA, USA) on a MacBook Pro laptop (Apple Inc., Thailand). A standardized photograph of an ideal esthetic smile according to the literature (gingival margins and tooth alignment, shape, size, symmetry, etc.) is composed of teeth, lips, and surrounding skin [4]. Therefore, other facial structures (eyes, nose, and chin) were cropped out to reduce confounding variables. Three lip thicknesses (thin (Tn)/medium (*M*)/thick (Tk)) and lipstick shades (orange (*O*)/pink (*P*)/red (*R*)) were obtained by editing the photograph using the Adobe Photoshop software. Three different tooth shade tabs (0*M*1/0*M*3/*A*1) from a commercial shade guide (VITA classical *A*1–*D*4 shade guide with VITA bleached shades, Ivoclar Vivadent Inc., USA) were photographed separately using a digital camera. The three different tooth shades (0*M*1/0*M*3/*A*1) were modified using the Adobe Photoshop software. Each photographic image (JPEG files) was saved and exported to prepare a photographic questionnaire with 27 photographs for presentation on a 10.5-inch tablet (iPads air3, Apple Inc., Thailand) (Figure 1).

The pilot study was performed by three cosmetic dentists to assess and improve the content validity and ease of understanding of the questionnaire and by 10 Thai individuals (not from the Thammasat University) to estimate the time required to complete the questionnaire and determine the repeat measure reliability (three smile photographs were randomly selected and repeated in the photographic questionnaire for the reliability assessment) and comprehensibility of the questions. The intraclass correlation coefficient (ICC) was calculated to estimate the intra-examiner reliability of the questionnaire.

### 2.3. Smile Attractive Perception Survey

**Figure 2:**
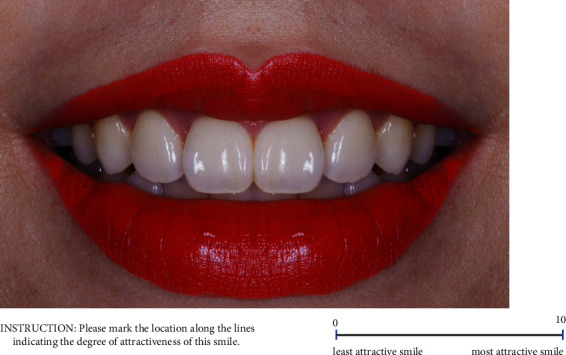
Example of a smile photograph on a page screen of the tablet scored using a 10 cm visual analog scale.

All participants who provided informed consent were recruited to complete the digital photographic questionnaire for rating the smile attractiveness perception in 27 smile photographs presented on a 10.5 inch tablet. Before starting Part 2 of the questionnaire, participants were asked to let their eyes rest by looking at a blue background. The tablet screen was set to 50% brightness and contrast for the rating duration. Participants rated each smile photograph on a page screen of the tablet in random order using a 10 cm VAS (Figure 2). A VAS score of 0, the lowest, indicated the least attractive smile, whereas a VAS score of 10, the highest, indicated the most attractive smile. Participants adjusted their scores on the tablet screen until they were satisfied. The smile in each photograph had to be scored within 10 s and returning to the previous photograph was not possible. The VAS score for smile attractiveness perception was determined by measuring the length in millimeters from the starting point on the left to the marked point on the right.

### 2.4. Statistical Analysis

All data were recorded in an excel sheet (Excel, Microsoft Corporation). The ICC was calculated from the VAS score for smile attractiveness perception in three repeated smile photographs taken by 10 examiners (not from Thammasat University). The median with interquartile range (I.R.) and mean with standard deviation (S.D.) of smile attractiveness perception (VAS score) for each data group were calculated. Statistical analysis was performed using SPSS version 16.0 (SPSS Inc., Chicago, IL, USA) at a significance level of ≤0.05. The Kolmogorov-Smirnov test was used to assess the data for variables that were not normally distributed. Non-parametricKruskal-Wallis and pairwise comparisons were used to compare the smile attractiveness perception of various lip thicknesses, lipstick shades, and tooth shades according to all participants and each rater group (dentists, laypersons, and dental and other students).

## 3. Results

The ICC for repeatability of the three smile photographs by 10 participants was 0.85, 0.85, and 0.91, which demonstrated good to excellent reliability of the response to the questionnaire.

**Figure 3:**
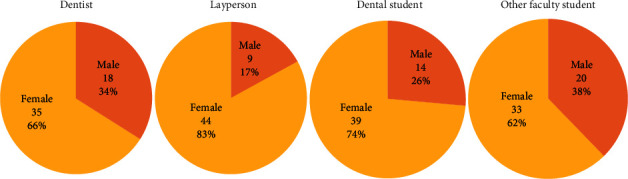
Percentage of males and females in each rater group.

A total of 212 raters responded to and completed the survey. In all rater groups, the number of females was higher than that of males (Figure 3).

The median (I.R.) VAS score for smile attractiveness perception was evaluated using a combination variable (lip thickness × lipstick color × tooth shade) in 27 photographs rated by all participants and each rater group, as shown in Table 1. The median VAS score for almost all photographs provided by the dental student group significantly differed from that provided by other groups. The photograph with *M*/*R*/0*M*1 was given the highest VAS scores for smile attractiveness perception by all participants and other faculty students while that with *M*/*R*/0*M*3 was given the highest VAS scores by dentists and laypersons. In contrast, the Tk/*R*/*A*1 photograph was given the highest VAS score by dental students, and the Th/*O*/*A*1 photograph was given the lowest VAS scores by all participants and other faculty students. Th/*O*/0*M*3, Th/*P*/*A*1, and Th/*P*/0*M*3 photographs were given the lowest VAS scores by dentists, laypersons, and dental students, respectively.

**Figure 4:**
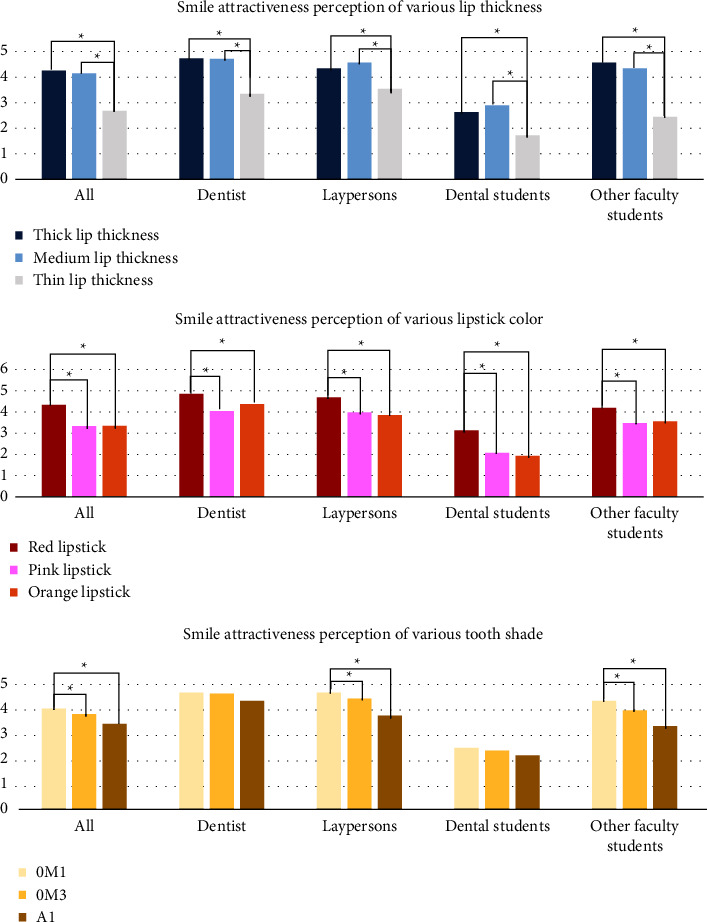
Bar chart of median visual analog scale scores for smile attractiveness perception as evaluated by each variable by all participants and each rater group. The asterisk bracket (^*∗*^) indicates significant differences between groups (*P* ≤ 0.05).

The median with I.R. and mean with S.D. of VAS score for smile attractiveness perception in smile photographs as evaluated by each variable (lip thickness, lipstick color, and tooth shade) by all participants and each rater group are shown in Table 2 and Figure 4.

Tk or *M* lip thickness was associated with more smile attractiveness than Th lip thickness. The *R* lipstick was found to provide a more attractive smile than the *P* and *O* lipsticks. The 0*M*1 tooth shade appeared to be the most attractive for laypersons and other faculty students, whereas no tooth shade (0*M*1, 0*M*3, or *A*1) affected the smile attractiveness perception of dentists and dental students.

## 4. Discussion

The number of cosmetic smile makeovers is increasing. Smile attractiveness is an important aspect of the face and affects a person's appearance [1, 14, 27]. A dentist has the opportunity to create an attractive smile based on the patient's perceptions and expectations. Many previous studies on smile attractiveness have focused on aspects such as the smile line, smile arc, gingival display, and buccal corridor [14, 16, 17]. However, no study has evaluated the influence of lip thickness, the application of a lipstick, and tooth shade. Therefore, this study focused on smile attractiveness perception of a woman's smile with various lip thicknesses, lipstick shades, and tooth shades by different rater groups using VAS scores.

Smile makeover planning is incomplete by considering only smile elements and the preferences of the treatment provider (dentist and dental student). It is necessary to know which smile characteristics are attractive to patients (other students and laypersons) to guide the design of an attractive smile for each individual. Dentists and laypersons have different esthetic perceptions [5, 13, 18]. In addition, dental and smile esthetic perceptions differ between dental and nondental students [19, 20]. Therefore, four rater groups, including dentists, laypersons, dental students, and other faculty students, were selected for this study.

The VAS score was used to rate the smile attractiveness perception because it is simple, scores can be obtained fast, and it has good to excellent reliability (ICC 0.85–0.91). The questionnaire was prepared according to the suggestions of the three dentists for the ease of understanding of participants. All bleached shades (0*M*1, 0*M*2, and 0*M*3) and the brightest natural (*A*1) shade are the current popular tooth shades in cosmetic dentistry for creating a more attractive smile. However, in this study, the VITA bleached shades with the highest and lowest brightness (0*M*1 and 0*M*3, respectively) and the brightest natural shade (*A*1) were selected, and the VITA bleached shade with moderate brightness (0*M*2) was excluded, following the suggestion of the three dentists who guided the questionnaire development to reduce the number of photographs and time spent in exploring the data obtained from each participant. Smile attractiveness perception is not known to differ significantly between raters of different genders [15–17, 21, 22]. Thus, this study collected the data from each rater group without considering the gender of the participants. However, face and mouth photography does not influence smile attractiveness perceptions [2]. In the literature, smile photographs included in questionnaires have included teeth, lips, and a minimal amount of surrounding skin to avoid the effects of facial structures on smile attractiveness [5, 14, 17, 23]. The set of 27 smile photographs used in this study was ordered randomly, and each photograph was rated within 10 s. Thus, the raters did not know what aspect was modified in each photo and did not rate in an incremental trend, which eliminated the bias of rating.

This study focused on smile attractiveness perception according to lip thickness, lipstick colors, and tooth shades as evaluated by all participants and each rater group (dentists, laypersons, dental students, and other faculty students). The results of this study revealed a significant difference in smile attractiveness perceptions among all rater groups for various lip thicknesses and lipstick colors. Furthermore, there were significant differences in the smile attractiveness perceptions among all participants, laypersons, and other faculty students according to tooth shades. However, the difference in tooth shade did not affect the smile attractiveness perception of dentists and dental students. The results of this study revealed that Tk and *M* lips were associated with more smile attractiveness than Tn lips. *R* lipsticks are more attractive than *P* and *O* lipsticks. The brightest tooth shade, 0*M*1, appeared to be the most attractive for laypersons and other faculty students. In contrast, tooth shades did not influence the smile attractiveness, according to dentists and dental students.

The results of this study were based on different rater groups. The VAS scores provided by dentists, laypersons, and other faculty students did not show statistically significant differences in almost any smile photo. The VAS scores by dental students for almost every photo were statistically lower than those by dentists, laypersons, and other faculty students. The dental students in this study were 3^rd^ to 6^th^ year students who had just begun to know the components of a beautiful smile. This result may be explained by the fact that dental students who are a part of the dental faculty are taught about the components of a smile during the academic curriculum, which may influence the needs and expectations of attractive smiles more than other rater groups.

Lip appearance conveys personal characteristics and attractiveness [13, 24]. The present results show that thicker lips positively influenced the smile attractiveness perception of all rater groups. This finding is consistent with the results reported by many researchers. McNamara et al. [6] and Rai et al. [25] found that Tk lips were an attractive feature and that lip thickness determined the smile pleasantness perceptions of orthodontists and laypeople. Moreover, Kar et al. [26] reported that fuller lips of women tended to be more attractive. Whereas, Dobke et al. and Paul et al. found that an individual's ethnic, cultural, and demographic backgrounds may influence attractiveness or beauty perception. Dobke et al. [27] found that fuller lips are preferred by Korean women, while thinner lips are preferred by Japanese women. Heidekrueger et al. [28] found that larger lips were preferred by Asian or non-Caucasian clinicians, while smaller lips were preferred by European and Caucasian clinicians. The preference of women for Tk lips may be explained by the results of Gunn et al. [29] and Ramaut et al. [30], who demonstrated that larger lips make women look younger and Tn lips occur during the lip-aging process in older people.

In the 21^st^ century, the use of lipsticks has consistently increased facial and smile attractiveness. Lip color is a major determinant of facial attractiveness and sex typicality [9]. Lipsticks are used to create more contrast between the lips and facial skin, which leads to enhanced perceived attractiveness [31]. Lipsticks are available in various shades that customers can choose for attractive enhancement. The results of our study showed that different lipstick shades influenced smile attractiveness perception: *R* lipstick was found to be the most attractive, while *P* and *O* were less attractive for all rater groups. This may be explained by the fact that, traditionally, the color *R* (blue-purple shaded undertones) is known to help in countering any yellowness of facial skin and teeth, making smile and face appear whiter and more attractive. Consistent with the results obtained by Kobayashi et al. [32], lip color has been known to affect facial skin color perception; reddish lips make the skin look lighter, and *O* lips make the skin look yellowish.

For smile reconstruction procedures, tooth shade selection has been challenged to create attractive smiles based on the perception of each person. Dentists usually choose bright tooth shades depending on the number of tooth reconstructions, skin color, eye color, and personal expectations [11, 33]. The present study found that brighter tooth shades tended to enhance smile attractiveness for all rater groups. Laypersons and other faculty students preferred Hollywood smiles with the whitest bleached shade (0*M*1), which is considerably more white than natural teeth, probably due to the influence of multimedia, emphasizing that the extreme whiteness of teeth makes the smile more attractive to the population [12, 34, 35]. Furthermore, most dental clinics promote whitening dental cosmetic treatment through advertisements and search engines [35]. Dentists and dental students preferred the natural bright shade (*A*1), which is not statistically different from Hollywood smiles (0*M*1 and 0*M*3) bleached shades. This may be explained by their education on beautiful smiles with naturally bright teeth. This finding is inconsistent with the study by El-Mourad et al. [36], who reported that younger dental students and laypersons preferred bleached shades due to insufficient professional knowledge and the effects of social media; in contrast, higher-year dental students tended to prefer a more natural tooth color.

Based on the results and limitations of this study, a proper understanding of lip appearances and tooth shades preferred by patients is essential to creatings an attractive smile. Several limitations of this study must be considered while interpreting the results. The data were collected from a static photograph of a woman's smile with limitations of location and variables (lip thickness, lip colors, and tooth shades) that cannot gauge dynamic emotional smile attractiveness and smiles in other ethnicities and culture, and did not consider the effect of other components of the smile, especially in the dynamic movement. Thus, several demographic backgrounds and variables that affect smile attractiveness should be studied further. Moreover, factors that influence the smile attractiveness of the male sex and LGBTQ+ community would also be interesting to study in the future.

## 5. Conclusion

The integration of lip and tooth characteristics affects the smile attractiveness in different groups of people. A smile with thick or medium-thick lips and a reddish tint was considered the most attractive by the entire rater group of participants. Laypersons and other faculty students preferred smiles with the whitest bleached shade (0*M*1). However, the perception of dentists and dental students did not show a significant difference between natural bright (*A*1) and bleached (0*M*1 and 0*M*3) tooth shades.

## Figures and Tables

**Table 1:** Median (interquartile range) visual analog scale scores for smile attractiveness perception as evaluated using a combination variable (lip thickness × lipstick color × tooth shade) by all participants and each rater group.

Variables (lip thickness, lipstick color and tooth shade)			All (*N* = 212)	Dentists (*N* = 53)	Laypersons (*N* = 53)	Dental students (*N* = 53)	Other faculty students (*N* = 53)
Tk	*R*	0*M*1	4.74 (3.81) cd	5.55 (3.57) Abc	5.30 (4.26) Ab	3.53 (4.08) Bb	4.36 (3.76) Bc
		0*M*3	4.93 (4.27) d	4.94 (3.84) Ac	4.88 (3.52) ABb	4.02 (4.19) Bbc	5.43 (4.25) Ac
		*A*1	4.80 (4.68) cd	5.41 (3.53) Ac	4.42 (5.50) Aab	4.58 (5.08) Ab	4.89 (4.32) Ac
	*P*	0*M*1	3.66 (3.74) b	4.40 (3.29) Aa	4.88 (3.98) Ab	2.29 (4.05) Bb	4.13 (4.01) Ab
		0*M*3	4.08 (3.65) b	4.43 (3.77) Ab	4.3 (3.12) Ab	2.37 (2.98) Bb	4.70 (3.81) Ac
		*A*1	3.76 (3.97) b	4.30 (3.23) Ab	3.83 (4.79) Aa	2.65 (3.61) Bb	4.00 (4.68) Ab
	*O*	0*M*1	3.43 (3.91) b	4.84 (3.84) Ab	3.36 (3.25) Aa	1.73 (2.57) Bab	3.98 (4.84) Ab
		0*M*3	3.69 (3.58) b	4.54 (3.28) Ab	3.34 (3.4) Aa	2.63 (4.08) Ab	4.34 (3.62) Ab
		*A*1	3.31 (3.99) d	4.64 (3.66) Ab	3.35 (3.62) Aa	1.97 (3.38) Bb	3.76 (3.36) Ab

*M*	*R*	0*M*1	4.94 (5.14) d	5.16 (3.39) Ac	5.20 (4.49) Ab	3.96 (3.68) Bb	5.45 (5.05) Ac
		0*M*3	4.84 (3.99) cd	5.6 (3.37) Ac	5.32 (4.48) Ab	3.35 (3.90) Bb	4.98 (4.57) Ac
		*A*1	4.28 (3.82) dc	4.68 (3.95) Ab	4.6 (3.57) Ab	3.41 (3.80) Bb	3.99 (4.31) Ab
	*P*	0*M*1	4.38 (4.21) c	4.47 (5.20) Ab	4.57 (4.22) Ab	3.36 (3.42) Bb	4.81 (5.01) Ac
		0*M*3	3.25 (4.54) b	3.38 (4.22) Aa	3.66 (4.32) Aa	2.33 (4.34) Ab	2.91 (4.66) Ab
		*A*1	3.98 (3.80) b	4.19 (2.95) Ab	4.54 (3.93) Aa	2.42 (4.09) Bb	4.19 (3.65) Ab
	*O*	0*M*1	4.61 (3.87) c	4.90 (3.12) Ab	4.98 (3.52) Ab	2.42 (3.51) Bb	5.13 (3.52) Ac
		0*M*3	3.78 (3.49) bc	4.50 (3.70) Ab	3.96 (3.12) Aa	2.95 (2.77) Bb	3.92 (4.15) Ab
		*A*1	3.10 (3.71) a	4.41 (4.10) Aa	3.45 (3.67) Aa	1.65 (2.08) Bab	2.86 (3.27) ABa

Th	*R*	0*M*1	3.14 (3.78) b	3.48 (3.95) Aa	3.74 (3.65) Aa	2.14 (2.85) Bb	2.89 (3.84) ABa
		0*M*3	3.25 (4.56) b	4.1 (3.86) Aa	4.43 (5.21) Aa	2.12 (3.42) Bb	2.89 (3.84) Ab
		*A*1	2.63 (3.68) a	3.49 (4.14) Aa	3.03 (3.53) ABa	1.6 (3.20) Cab	2.23 (2.84) Ba
	*P*	0*M*1	2.54 (4.05) a	3.09 (3.93) AB	3.42 (4.66) AC	1.73 (2.76) D	2.53 (4.98) BCD
		0*M*3	2.37 (4.00) a	2.93 (3.72) Aa	3.49 (4.21) Aa	1.11 (1.96) Ba	2.37 (4.14) Aa
		*A*1	2.32 (3.48) a	3.01 (3.39) Aab	3.00 (3.40) Aa	1.3 (2.86) Bab	2.03 (2.69) ABa
	*O*	0*M*1	3.20 (3.88) a	3.61 (3.95) Aa	3.83 (3.74) Aa	1.84 (2.67) Bab	3.25 (4.25) Aa
		0*M*3	2.47 (3.91) ab	2.85 (4.56) ABab	3.96 (3.12) Aba	1.65 (2.05) Cab	2.37 (3.67) Ba
		*A*1	2.19 (3.86) a	3.23 (3.90) Aab	3.53 (4.06) Aa	1.3 (2.36) Bab	1.88 (3.66) Ba

Different uppercase and lowercase letters demonstrate statistically significant difference within the same row and the same column, respectively. Tk, thick; *M*, Medium; Th, thin; *R*, Red; *P*, Pink; *O*, Orange; 0*M*1, 0*M*3, and *A*1, tooth shades.

**Table 2:** Median (interquartile range) and mean (standard deviation) of the visual analog scale score for smile attractiveness perception as evaluated by each variable (lip thickness, lipstick color, and tooth shade) by all participants and each rater group.

Rater group	Variable								
	Lip thickness			Lipstick color			Tooth shade		
	Thick	Medium	Thin	Red	Pink	Orange	0*M*1	0*M*3	*A*1
All	4.26 (4.18)	4.15 (4.00)	2.67 (3.95)	4.33 (4.28)	3.34 (4.02)	3.36 (3.85)	3.95 (4.13)	3.73 (4.18)	3.36 (4.02)
	4.32 (2.68)	4.28 (2.62)	3.29 (2.60)	4.45 (2.72)	3.75 (2.68)	3.69 (2.56)	4.15 (2.70)	4.00 (2.71)	3.73 (2.60)
	*A*	*A*	*B*	*A*	*B*	*B*	*A*	*B*	*B*

Dentists	4.73 (3.81)	4.71 (3.81)	3.34 (3.90)	4.85 (4.01)	4.04 (3.54)	4.36 (3.84)	4.58 (3.77)	4.54 (3.90)	4.25 (3.76)
	4.82 (2.48)	4.77 (2.35)	3.67 (2.44)	4.49 (2.47)	4.07 (2.52)	4.24 (2.37)	4.54 (2.46)	4.41 (2.48)	4.30 (2.50)
	Aa	Aa	Ba	Aa	Ba	Ba	Aa	Aab	Aa

Lay persons	4.34 (3.92)	4.57 (3.77)	3.53 (3.98)	4.69 (4.33)	3.98 (4.06)	3.85 (3.47)	4.58 (3.88)	4.34 (3.90)	3.68 (3.93)
	4.56 (2.64)	4.60 (2.66)	3.99 (2.78)	4.80 (2.74)	4.23 (2.73)	4.12 (2.60)	4.65 (2.72)	4.47 (2.74)	4.03 (2.62)
	Aa	Aa	Ba	Aa	Ba	Ba	Aa	Ba	Ba

Dental student	2.63 (3.98)	2.89 (3.66)	1.71 (2.58)	3.12 (3.98)	2.08 (3.37)	1.93 (3.30)	2.42 (3.06)	2.31 (3.69)	2.12 (3.71)
	3.32 (2.60)	3.25 (2.36)	2.33 (2.10)	3.59 (2.59)	2.72 (2.31)	2.59 (2.18)	3.07 (2.42)	2.94 (2.41)	2.89 (2.38)
	Ab	Ab	Bb	Ab	Bb	Bb	Ab	Ac	Ab

Other faculty student	4.57 (4.19)	4.34 (4.20)	2.44 (3.85)	4.19 (4.61)	3.47 (4.48)	3.55 (3.95)	4.26 (4.51)	3.89 (4.77)	3.27 (4.02)
	4.57 (2.75)	4.49 (2.80)	3.85 (2.72)	4.45 (2.86)	3.99 (2.87)	3.80 (2.71)	4.35 (2.86)	4.18 (2.91)	3.70 (2.67)
	Aa	Aac	Bc	Ac	Ba	Bc	Aa	Ba	Bc

Different uppercase and lowercase letters demonstrate statistically significant difference within the same row of each dependent variable (lip thickness, lipstick shade, and tooth shade) and the same column, respectively.

## Data Availability

The data supporting this research article are available from the corresponding author or first author on reasonable request.
